# Supplementary material for: Accuracy of preferred language data in a multi-hospital electronic health record in Toronto, Canada
Source: PLOS Digit Health. 2025 Sep 3;4(9):e0000999. doi: 10.1371/journal.pdig.0000999 (PMC12407390; doi:10.1371/journal.pdig.0000999)
Supplement: Attachment — Submitted filename: Response to Reviewers_BABEL.docx [file pdig.0000999.s001.docx]

Dear Editor-in-Chief Dr. Celi,

Thank you for the opportunity to revise and resubmit our manuscript entitled, "Accuracy of preferred language data in a multi-hospital electronic health record in Toronto, Canada." We include in this letter detailed responses (R) to the *editor and reviewer comments (C)*. New or substantially adjusted text appears in red. We will separately submit tracked changes and clean versions of the manuscript. We edited the Discussion to reduce its length, after incorporating the Reviewer suggestions.

**Reviewer 1**

*C1: The authors should provide more details on the prospective audit’s sampling strategy. It would help to clarify whether patients were selected consecutively or randomly during the audit period and whether any inclusion/exclusion criteria were applied. The manuscript notes that most audited patients were from internal medicine wards – if the audit was limited to certain services or a short time frame, discuss how that might affect generalizability. Ensuring the reader understands how the 323 patients were chosen (and if any patients approached declined participation or were unavailable) will bolster confidence that the sample is representative of the hospital populations.*

R1: We audited the internal medicine ward, nephrology ward, and intensive care unit at each hospital. Patients were selected consecutively. We excluded only those patients whom we were unable to determine whether their preferred language was English or not. We have added these details to the methods section:

“The only inclusion criterion was admission to the internal medicine ward, nephrology ward, or intensive care unit. The only exclusion criterion was inability to determine whether their preferred language was English. One investigator (CF) prospectively interviewed consecutive patients admitted to the intensive care unit, internal medicine ward, and nephrology ward across the three hospitals.”

We have updated our limitation section to include sampling specific hospital services and over a short time period:

“Generalizability of this audit may be limited by the single-month duration, sampling of specific hospital services, potential site-level variation, and the unique linguistic diversity of Toronto’s population.”

If patients were unavailable their substitute decision maker (SDM) was contacted. No patients declined participation. We have amended the result section:

“Between June 17, 2024, and July 19, 2024, we identified 349 patients across 3 hospitals, of whom 26 were excluded because we could not contact them or their SDM. No contacted patients or SDMs declined participation.”

*C2:* *The handling of patient non-response needs more explanation. Nearly 41% of the interviews were conducted with substitute decision-makers, which is a pragmatic solution for incapacitated patients but could introduce uncertainty in the “true” preferred language. The manuscript should clarify if any eligible patients could not be interviewed at all and how those were handled (e.g., excluded or replaced). It would also be worthwhile to comment on whether the agreement between EHR and the interview differed when the information came from a substitute decision-maker versus the patient directly. If data permits, consider a brief analysis or* comment *in the Discussion on whether proxy responses might affect the accuracy.*

R2: We acknowledge that the use of substitute decision makers can introduce uncertainty in the true preferred language. We have highlighted this point in the limitations:

“For patients who were unable to provide a response, we relied on substitute decision makers to indicate preference, whose response may differ from the patient’s true response.”

Patients who could not be interviewed and had SDMs whom we could not reach after 2 attempts were excluded (see R1).

We investigated how concordance differs between interview responses elicited from patients and SDMs. We found that the prevalence of interview-based non-English preferred language was higher when responses were elicited from SDMs, but there were no differences in accuracy.

To the Methods, we added: “As a sensitivity analysis, we repeated the primary analysis in subgroups defined by whether we obtained interview-based preference from the patient or their SDM.”

To the Results, we added: “Interview-based non-English language preference was less common when the patient was the source compared to when the SDM was the source (50/191 [26%] vs 58/132 [44%], absolute difference 17.8% [CI 4.2% to 25.2%, p < 0.01]). Whether interview-based preferred language was obtained from patients or SDMs, accuracy was similar (169/181 [88%] vs 109/132 [83%], p = 0.13) and Cohen’s kappa was similar (0.68 vs 0.67).”
*C3: The use of sensitivity, specificity, PPV, and NPV to quantify the accuracy of EHR language data is appropriate for the dichotomized outcome (non-English vs. English preference). It may be useful to report an overall agreement statistic beyond simple percentage agreement; for instance, a Cohen’s kappa could contextualize the 86% agreement by accounting for the large proportion of English-preference patients. Additionally, please clarify the statistical test used for comparing the prevalence of non-English preference between interviews and EHR (the 9.6% absolute difference). A McNemar’s test would be appropriate for this paired comparison – if that was used, stating it explicitly would be helpful for readers.*

R3: The Cohen’s kappa was 0.69 (CI 0.608 to 0.772), including a relatively low expected probability of agreement (0.54) which reflects the moderately high prevalence of non-English preference. We have added the Cohen’s kappa to the results.

We added the following to the Methods: “In the primary analysis, we calculated percentage agreement and Cohen’s kappa; …” and to the Results: “The interview-based and EHR-based preferred language agreed in 278 (86%) of patients and Cohen’s kappa was 0.69 (CI 0.61 to 0.77) (Table 3).”

The confidence interval for the absolute difference was based on a t-test comparing proportions. We added the following to the Methods:

“We compared the absolute difference in percentage of patients preferring a non-English language between interview-based and EHR-based methods using a two-sided test of proportions.”

As the reviewer suggests, a McNemar test can be used to assess whether discordant pairs are more likely to have a true preference for English or non-English language. We have added the following to the Methods: “We used a McNemar’s test to assess whether discordant responses were more likely to prefer a non-English language.” We then added this to the Results: “The true preference in patients with discordance was more likely to be non-English language (OR 5.43 [CI 2.43 to 12.17], p < 0.001).”

*C4: The manuscript should expand on the modeling decisions to improve transparency. The authors could specify the prior distributions used for parameters in the Bayesian model (even if default weakly-informative priors were used) and note whether any prior sensitivity analysis was performed. Additionally, consider commenting on model diagnostics: for example, whether the Markov Chain Monte Carlo converged well (R-hat values ~1, adequate effective sample sizes) – even a brief mention would reinforce confidence in the Bayesian results. Finally, given the multi-hospital setting, the decision to model hospital and service as fixed effects (with odds ratios for each) rather than random effects is acceptable, but it may help to briefly justify this choice (e.g., treating the three specific hospitals as fixed factors of interest due to known policy differences).*

R4: We used skeptical prior distributions with no sensitivity analyses. We now include the rationale for modeling hospitals as fixed effect. The sentences added to the Methods section are: “Hospitals were modeled as fixed effects because there were only 3, with known differences in the distribution of languages. Prior distributions for fixed effects were skeptical (normal(0, 0.5) in log-odds space).”, and “We did not perform sensitivity analyses incorporating alternative prior distributions.”, and “Models were fit in R using the brms package, with 4 chains, each with 1000 warmup and 2000 model fit iterations.”

The model diagnostics were reassuring:

We summarized this information in the results by adding the following sentence: “Model diagnostics were reassuring, with all R-hat values equal to 1, no divergences, and effective sample sizes all greater than 2500.”
*C5: It might be useful to provide some additional insight (perhaps in a Supplement) into language-specific accuracy if possible – for instance, were there any notable trends such as certain languages consistently being missed by the EHR?*

R5: We agree that language-specific accuracy is important. Unfortunately, our sample size limits inference about specific languages, as demonstrated by the wide credible intervals for the language-specific coefficients in our Bayesian analysis (Figure 2). We have maintained the following statement in our limitations: “Our sample size limits strong conclusions regarding accuracy according to non-English preferred language…”
*C6: The current results indicate wide credible intervals for individual language effects, suggesting no statistically significant outliers. However, the authors need to ensure that the text does not over-interpret any single language’s result given the small sample sizes. The authors may also consider explicitly stating that very low-frequency languages were included in the model via random effects, which helps borrow strength from the larger groups – this could be highlighted as a benefit of the Bayesian multilevel approach.*

R6: Thank you for the reminder to avoid over-interpretation of the data. We adjusted the first sentence of the final paragraph of the Results which now reads: “There was minimal variation in odds of correct identification across non-English preferred languages.” The caption of Figure 2 still includes the statement: “Note that no languages are convincingly associated with higher or lower probability of correctly identifying non-English preference.”

We have added a comment to highlight the strength of Bayesian multilevel modeling mentioned by the reviewer. “In addition, a multilevel approach clustering by preferred language allows low-frequency languages to be included in the regression, without ignoring broad trends across non-English languages or collapsing them into an “Other” category.”

**Reviewer 2**

*C7: It may be helpful to briefly explain why ICU, nephrology, and internal medicine services were specifically chosen for inclusion in the study. This additional context would allow readers to better assess the generalisability of the findings to other hospital services or patient populations.*

R7: We have revised a portion of the methods to include a rationale for the service choice as follows:

“These services were chosen due to the role language-appropriate care plays in each service. Patients found on all three services can have complex, multi-system conditions that require frequent communication between patient and clinical team, and important decisions regarding goals of care and other interventions. Together, these services make up a large proportion of inpatients across the three hospital sites.” *C8: Although the introduction is well written, it may benefit from highlighting Toronto’s well-known linguistic diversity. This would further underscore the relevance and uniqueness of the setting and strengthen the rationale for studying preferred language accuracy in this particular health system.*

R8: We have revised the last paragraph of the introduction to mention Toronto’s linguistic diversity as follows:

“Identifying preferred language is especially relevant in Toronto, Canada, where 42.5% of the population have a mother-tongue other English or French (7).”

*C9: In Table 2, changing “Preferred Spoken Language” to “Interview-Based Preferred Language” may enhance clarity.*

R9: We have amended the title in table 2 as follows:

*“*Interview-Based Preferred Language*”*

*C10: Consider adding more detail on how frontline staff collect and enter language preference data during registration, as this could give further insight into variation across hospitals.*

R10: We have added more information on the preferred language process, and moved this information from the Discussion to the Method’s section, as follows:

“At the health network we studied, asking about preferred language is routine and mandated during patient registration for appointments or hospital admissions. The patient’s preferred language is then entered into the EHR.” *C11: Consider noting the short study period (approximately one month) as a potential limitation and whether seasonal admission patterns could influence generalisability.*
R11: We have updated the limitations portion to include the seasonal admission patterns effect on generalizability as follows:

“Generalizability of this audit may be limited by the single-month sampling period, sampling of specific hospital services, potential site-level variation, and the unique linguistic diversity of Toronto’s population.”

**Reviewer 3** 
*C12: The sentence that states “Language concordance, via shared language proficiency or interpretation” should be rephrased for accuracy. Interpreter-mediated care is not language-concordant. There is still a discordance between the clinician and patient during interpreter-mediated care. However, it is language-appropriate because the interpreter serves as the linguistic conduit to negotiate meaning between the patient and clinician. The authors could consider saying “Language-appropriate care, via shared language proficiency or interpretation” or could consider other adjustments for clarity.*

R12: Thank you for the important point regarding terminology. We have adjusted language-concordant to language-appropriate in the introduction and other places, and added a citation to Ortega et al doi:10.1001/jamanetworkopen.2023.24485. For example, in the introduction: “Language-appropriate care, where a patient receives care in their preferred language for healthcare communication via shared fluency or professional interpretation, can help address potential disparities.”
*C13: The authors explain prior related studies on the accuracy of preferred language data and explain the gap that this study aims to address. It seems to me that there are other things that distinguish this study from the two that were cited, references 8 and 9. For example, ref 8 is a US study and therefore reflects a linguistically different population compared to Canada. It may be worth pointing this out to add to this study’s contribution to the literature.*
R13: The 2^nd^ paragraph of the introduction has been updated to note the US localization of the reference:

“Another study conducted in an outpatient setting in the USA found that the EHR accurately identified preferred language in two-thirds of Spanish-speaking patients.(9)”
*C14: However, if the patient had a non-English language preference, how was the question asked? Was there a protocol for when an interpreter was used to ask the question (e.g., based on what was documented in the EHR? Something else?). What types of interpreters were used to ask the question? If interpreters were not used, then what other approaches were used to ensure the question and response were communicated accurately?*

R14: The question on language preference was only asked in English. If a patient was unable to understand the question about language preference due to limited English proficiency or otherwise, a SDM was contacted. All SDMs were able to provide a language preference for their patients. The methodology description has been updated to improve clarity as follows:

“A patient was considered unavailable if they were not present at the time the interviewer passed by their room. A response was considered unable to be elicited if the patient was incapacitated, intubated, or unable to understand the question on language preference. If the patient was unavailable at the time of interview, or a response could not be elicited, a substitute decision maker (SDM) was contacted…” *C15: The methods state that if “a response could not be elicited”, a substitute decision-maker was contacted. What does it mean for the response to not be able to be elicited? Was it based on the patient’s mental status, language/inability to communicate, refusal, or a combination of reasons? Some explanation here would be important.*

R15: Thank you for highlighting the need for clarification on this point. We believe that R14 above should address this concern.  *C16: The authors should move the first several sentence of the discussion into the results.*

R16: We have revised these sentences to reduce repetition and provide greater focus on discussion:

“In this prospective point-prevalence audit, we interviewed 323 patients across 3 hospitals and found that the EHR correctly captured patient preferred language in 86% of all patients, and 69% of patients who preferred a non-English language. Accuracy for patients who preferred non-English languages was worse for patients who were younger or male, varied by hospital site and most responsible service, and did not vary by non-English preferred language. These results have implications for research investigating differences according to preferred language, highlight an opportunity to improve preferred language data, and underscore the challenges of providing language-appropriate care in linguistically diverse patient populations.”

*C17: The discussions should begin with the major findings summary but without repeating the findings themselves that were already presented in the results.*

R17: We have restated the study design and main findings to highlight these for readers, as mentioned in R16, but have tried to limit repetition.
*C18: Is there also room to discuss the dynamic nature of language preference? Could this be part of the reason that there is a discrepancy, and that some amount of discrepancy in this data point may be unavoidable? My meaning is that depending on the context, a patient may have differing preferences with regards to the language they would want to use in a particular health care encounter. For example, if a person has basic English skills but generally prefers their health care in Cantonese, is it possible that this person would choose Cantonese if they had a Cantonese speaking doctor but English if they had an English-speaking doctor who would need to use an interpreter if they were to hold the encounter in Cantonese? Some more nuanced discussion of the dynamic nature of language preference and impact of multilingualism in the community would be warranted and useful.*

R18: Thanks for this thoughtful point. We added the following to the limitations:

“We treated language preference as fixed across all healthcare encounters, which may not reflect reality for some patients who are comfortable in English for some encounters but would prefer another language for other encounters.”

And:

“Dichotomizing language preference into English and non-English groups did not account for the dynamic nature of language preference or for patients who might be multilingual.”

*C19: Perhaps related to my latter point, what is the authors’ interpretation of the finding that older patients’ language preference was more reliably documented in the EHR compared to younger patients? I can think of several possibilities, and it may be useful for the authors to share some potential thoughts and perhaps a future research agenda that could test the potential factors involved.*

R19: We agree with the reviewer that several factors could explain the finding that older patients’ language preference was more reliably documented. The extent to which this finding will generalize across other health systems or contexts is unclear. We think that involvement of other family members with greater English proficiency is the most likely explanation, and have added the following to the discussion: “The reasons for lower accuracy among male compared to female patients and younger compared to older patients are not available in our data, but could relate to the involvement of additional family members, and represents an opportunity for further inquiry.”

*C20: Why do the authors think the accuracy rate of the language data field varied across languages? Could there be cultural, historical, or political factors that affect how the data is reported/collected or how patients respond?*

R20: There were no evident differences in accuracy rate by language, likely due to wide margins of uncertainty in measuring language-specific effects.

*C21: The authors could include a call for the collection of language proficiency data about health care professionals, which is also needed to conduct language concordance research. See, for example, Ortega et al. JAMA Network Open:* [*https://jamanetwork.com/journals/jamanetworkopen/fullarticle/2807144*](https://jamanetwork.com/journals/jamanetworkopen/fullarticle/2807144)*.*

R21: We have added a sentence to the discussion with the above citation: “Data on fluency of healthcare providers is also important.” *C22: The authors provide some practical recommendations in the discussion, such as the tips for registration staff. I would welcome a table or chart that summarizes the key strategies/recommendations the authors have for health systems to implement a better system for collecting language preference.*

R22: We agree that such a chart could be a helpful tool. However, our data do not inform on the effectiveness of different strategies or recommendations. In this manuscript, the primary focus is on reporting the results of our audit, and we have already included 3 tables and 2 figures. For now, we have deferred making this table, but we agree with the reviewer that it would be a valuable contribution within a commentary or other manuscript type.

*C23: Are there quality improvement/audit metrics that should be used, and if so, with what frequency, to ensure the accuracy of the EHR language preference? How should the language preference question be asked by clerks registering patients?*

R23: Accuracy of the language data can serve as a metric to guide quality improvement focused on the quality of language data. We think the frequency should probably be guided by the goals and interventions planned as part of a quality improvement project, and are difficult to dictate without that additional context. Regarding the question that clerks should ask, we have added the following to the Discussion: “Front-line administrative staff can ask about language preference in a standardized manner, using a question similar to the one used in this audit.(26)”

*C24: It is unclear what the authors mean by this sentence: “We did not account for bilingual or moderately fluent individuals.”*

R24: We have updated the initial part of the limitations to improve clarity as follows:

“Dichotomizing patients’ language preference into English and non-English groups did not account for the dynamic nature of language preference or patients who might be multilingual.” *C25: Additional limitations should be considered such as how generalizable (or not) these results might be in other types of hospitals/health systems, cities, countries, etc.*

R25: We have added additional limitations to the end of the paragraph to include the following comments about generalizability:

“Generalizability of this audit may be limited by the single-month sampling period, potential site-level variation, and the unique linguistic diversity of Toronto’s population.”
*C26: Consider adding suggestions for future study that could address remaining questions.*

R26: We have added some suggestions for future research in the Discussion paragraph that concerns differences according to patient and health system variables and include some at the Conclusion of the paper: “Next steps include analyzing the process of data collection and entry to improve data quality, and investigation of differences in care processes or outcomes according to preferred language.”
