# Supplementary material for: Accuracy of preferred language data in a multi-hospital electronic health record in Toronto, Canada
Source: PLOS Digit Health. 2025 Sep 3;4(9):e0000999. doi: 10.1371/journal.pdig.0000999 (PMC12407390; doi:10.1371/journal.pdig.0000999)
Supplement: Attachment — Submitted filename: Response to Reviewers_R2.docx [file pdig.0000999.s002.docx]

Dear Editor-in-Chief Dr. Celi and Section Editor Dr Kwak,

Thank you for the opportunity to revise our submission, “Accuracy of preferred language data in a multi-hospital electronic health record in Toronto, Canada.” We have included tracked-changes and clean versions of the manuscript, along with this letter where we respond to editor and reviewer feedback.

**Editor comments:**

*C1. Thank you for your thoughtful revision. I have one additional suggestion regarding the interpretation of the “preferred language ≠ English” variable. In English-speaking healthcare settings, this variable can represent a complex mix of factors beyond limited English proficiency—including bilingualism, cultural identity, administrative choices during intake, and the influence of available language services. Patients may select a non-English language due to family context or offered support, not necessarily because of communication difficulty.*

R1. Thank you for this insightful suggestion. We have added the following sentence to the limitations section to incorporate the elements of the feedback not already included in the limitations: “Language preference in an English-speaking healthcare setting is driven by a complex mix of factors in addition to English proficiency, including cultural identity, perceived appropriateness of requesting interpretation, and the logistical availability of language services.”

*C2. Additionally, “language comfort” is a subjective construct that is difficult to assess uniformly, and the absence of complementary indicators such as interpreter use, health literacy, or communication satisfaction may limit interpretability.*

R2. Great point. We added the following sentence to the limitations section in the Discussion: “In addition, language preference is a subjective construct, so the absence of complementary data such as interpreter use, health literacy, or communication satisfaction with respect to the health care team limit the interpretability of the results.”

*C3. For these reasons, I encourage you to acknowledge these limitations in the Discussion or Limitations section to help contextualize the role of language preference and avoid overgeneralizing this as a proxy for communication vulnerability.*

R3. We added the following sentence to the Limitations section in the Discussion: “This means that language preference is only a limited proxy for communication vulnerability.”

**Reviewer 2 comments:**

*C4. Thank you for addressing the comments I raised. Just a minor correction (Line 26-27): there appears to be a grammatical issue in the revised sentence—“have a mother-tongue other English or French.” To improve clarity and correctness, consider changing it to “have a mother tongue other than English or French.” Also, the hyphen in “mother tongue” is unnecessary.*

R4. Thanks for catching this, we have corrected the error.
